# Cross-cultural adaptation and validation of the Lithuanian version of the Spine Functional Index

**DOI:** 10.1371/journal.pone.0299719

**Published:** 2024-03-13

**Authors:** Giedrė Vaičienė, Kristina Berškienė, Vidmantas Zaveckas, Vilma Tamulionytė

**Affiliations:** Department of Sports Medicine, Lithuanian University of Health Sciences (LSMU), Kaunas, Lithuania; Cairo University, EGYPT

## Abstract

**Background:**

Low back pain is one of the most frequent medical problems caused by different factors. It is important to evaluate low back pain by choosing the best suited tool for the specific spine condition and pain severity. The Spine Functional Index (SFI) is a relatively new physical functioning-related questionnaire that can be used to assess different aspects of daily activities and movements. The purpose of this study was to cross-culturally adapt the SFI for the Lithuanian language and to determine its psychometric properties of validity, reliability, construct stability, internal consistency and factor structure.

**Methods:**

The study was designed as a two-stage observational study. Double forward and backward translations of SFI were performed for cultural adaptation for the Lithuanian language. For evaluation of psychometric properties, 125 participants with non-specific low back pain (duration of symptoms ≥ 6 weeks) rated their pain using Numeric Rating Scale, completed the Lithuanian version of SFI and Oswestry Disability Index. In 3 to 7 days all participants completed Lithuanian version SFI for the second time. The full sample was employed to determine internal consistency, test-retest reliability, construct stability, measurement error, construct validity and factor structure.

**Results:**

There was good internal consistency and reliability with Lithuanian version of SFI as Cronbach’s α = 0.92 and r = 0.82. Spearman-Brown coefficient was 0.97 representing good construct stability. Measurement error from standard error of the mean (SEM) was 6.96, from Minimal Detectable Change (MDC) was 16.24. Construct validity between the Lithuanian version of SFI and Oswestry Disability Index was excellent (ρ = 0.83), and good between the SFI and Numeric Rating Scale (ρ = 0.55). The factor analysis demonstrated a one-factor solution explaining 35.04% of total variance.

**Conclusion:**

Lithuanian version of SFI is a new reliable and valid instrument for functional evaluation of back pain in Lithuanian speaking patients.

## Introduction

Non-specific low back pain (LBP) affects patients of all ages. It is a disabling musculoskeletal condition and a leading contributor to disease burden worldwide [1,2]. According to the data of the Lithuanian Department of Statistics, the prevalence of lower back disease or other chronic back problems in 2019 was 30.3%. Comparing age groups, the prevalence was 24.9% in the 15–64 age group, and 48.2% in the 65+ age group [3]. LBP is one of the most frequent medical conditions that leads to lost worktime thus causing significant economic impact [4].

LBP can be caused by a variety of factors; it is therefore categorized as a symptom, rather than a disease. Structural cause of LBP cannot be confirmed in up to 85% of cases. In these circumstances LBP is defined as non-specific [1,5].

Various clinical tests, scales, and questionnaires are used to evaluate non-specific LBP in clinical practice as well as in scientific studies [6–11]. Patient-Reported Outcome (PRO) measures are the tools or instruments (e.g., questionnaires and scales) that are used to measure patients’ health status directly reported by the patient response [12]. It is an efficient and convenient tool assessing disability; therefore, it should be used for evaluation of treatment effectiveness [13]. PRO measures are essential in patient-centered care for LBP, especially when applying non-pharmacological interventions. This assessment is important to ensure the patients’ motivation, active involvement, self-management and adherence [14]. Furthermore, quantification of patients’ feedback can assist therapists while evaluating the function and symptoms as well as the intervention outcomes [14,15]. Various PRO measurements have certain advantages and limitations. It is important to choose the tool that is best suited for the specific condition and pain severity in medical practice as well as in research studies [16].

Oswestry Disability Index (ODI) is considered a “gold standard” for measuring functional status and disability associated with LBP [17,18]; it is a valid, reliable, and responsive tool, the most sensitive for evaluation of patients with persistent severe disability. The disadvantages of ODI includes weaker sensitivity in patients with mild to moderate disability [15] and absence of questions related to difficulty changing the posture such as from sitting to standing [19]. The Spine Functional Index (SFI) is another PRO tool which evaluates spine-related patient status and its change over the time. It was recently proposed and validated [20] and since then gained reputation as a valuable instrument with acceptable psychometric properties and strong practical characteristics in patients with various degrees of back pain severity [20–26].

To our knowledge, ODI is the only physical functioning-related questionnaire that is translated, cross-culturally adapted, and widely used in Lithuania [27–30]. For the purpose to complement evaluation set of physical functioning of patients with low back pain we aimed to translate and cross-culturally adapt the SFI into Lithuanian, and to determine its psychometric properties of validity, reliability, internal consistency and factor structure. Furthermore, we aimed to test how this questionnaire reflects the subjective functional status in a group of young people with mild to moderate or no LBP.

## Materials and methods

### Design

The study was designed as a two-stage observational study. The study involved participants who were patients from the Kaunas Clinics of the Lithuanian University of Health Sciences, as well as from three private clinics in Kaunas. During Stage 1 translation to Lithuanian language and cross-cultural adaptation of the SFI was performed. Stage 2 involved evaluation of the psychometric properties of the Lithuanian version (SFI-LT) and a pilot testing of associations between the SFI-LT and the pain characteristics in a group of young subjects.

### Ethical statement

The study was conducted according to the principles of the Declaration of Helsinki and approved by Kaunas Regional Biomedical Research Ethics Committee (No. BE-2-38). All participants were introduced to the study purpose, design, and signed an informed consent.

### Stage 1. Translation and cross-cultural adaptation

Permission to start the translation process was obtained from the copyright holder–Elsevier and Mapi Research Trust–the provider of Clinical Outcomes Assessment.

The SFI translation and cultural adaptation was performed according to the guidelines recommended by the International Society for Pharmacoeconomics and Outcomes Research (ISPOR) [31] and by the Mapi Research Trust [32].

**Fig 1:**
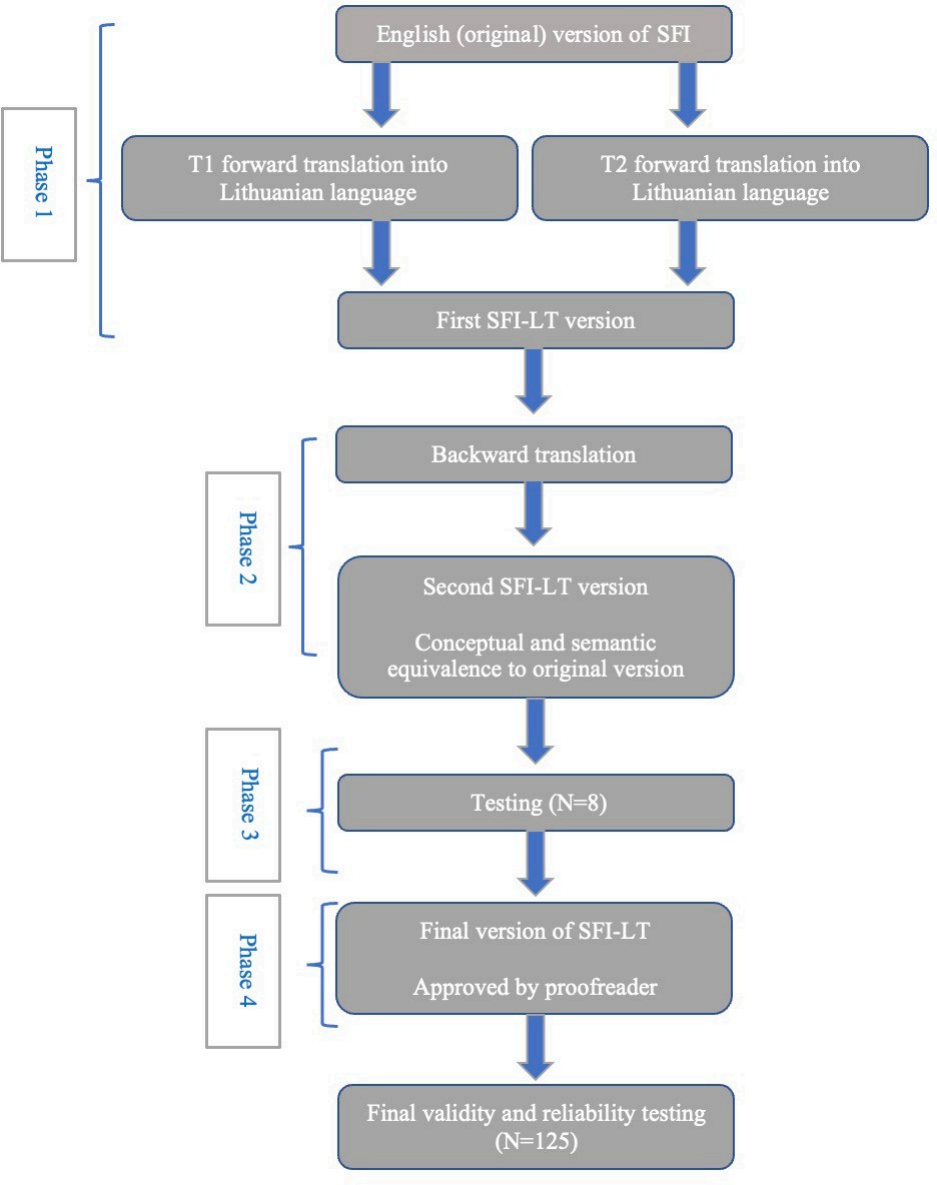
Flow chart of Spine Functional Index translation and cross-cultural adaptation process. T1 –translator 1, T2 –translator 2, T3 –translator 3, SFI–Spine Functional Index, N–number of participants, SFI-LT–Spine Functional Index-Lithuanian version (modified from [16] under a CC BY license, printed with permission from Elsevier, original copyright 2019).

The translation and cultural adaptation process followed 4 phases (Fig 1).

Phase 1: Forward translation. Two professional translators, both native Lithuanian speakers who specialize in medical translation (T1, T2), independently translated the questionnaire. Consensus of forward translation was reached and considered being equivalent to the original English language questionnaire. Issues regarding transliteration were documented.

Phase 2: Backward translation. Backward translation was carried out by a professional translator (T3), a native English speaker who is bilingual in Lithuanian, who had no access to the original version of the questionnaire. Comparison of the backward version with the original version was performed by the local coordinator. There were only a few translation issues due to linguistic intricacies of both languages. To avoid common errors in sentence construction in Lithuanian language we made minor changes in wording and used synonyms.

Phase 3: Testing. Eight subjects, five women and three men with chronic LBP, who are native Lithuanian language speakers were interviewed. Mean age of the subjects was 44.4 years (range of age 29–87). They did not encounter any difficulties understanding and filling in the consensus version of the forward translated questionnaire, nor asked for any assistance. As a result, we accepted consensus version of SFI-LT as the final version.

Phase 4: Proofreading of the third version of the questionnaire was done by a native Lithuanian proficient in English. No typographical, spelling or grammatical errors were found.

### Stage 2. Psychometric investigation

Patients meeting the inclusion criteria at a university hospital and three private clinics in Kaunas were enrolled in the study. They were asked to complete the SFI-LT and ODI-LT questionnaires and evaluated their pain intensity using 11 point Numeric Rating Scale (NRS). Patients were asked to complete SFI-LT questionnaire for a second time after 3 to 7 days for the purpose to evaluate the test-retest reliability.

To ensure accurate and comprehensive data collection, assistance was made available to all participants for any queries or clarifications regarding the questionnaire items.

#### Inclusion/Exclusion criteria

The inclusion criteria: age > 18 years, duration of LBP for at least 6 weeks, native speakers of Lithuanian. Exclusion criteria: identified structural causes of the spine (e.g., facet osteoarthritis, herniated disk, fracture, etc.), inflammatory disease, neurological disease, or any metabolic disorder that may affect low back area, pregnancy, and use of analgesics or myorelaxants in the last 12 hours.

#### Study instruments

*The Spine Functional Index*. The SFI is a single facture structure, 25-item questionnaire which was used to assess the spine-related subjective functional status on everyday activities. The items of the questionnaire include various questions about daily life activities such as the ability to take care of oneself, ability to rest, socialize, work or move. This questionnaire has three response options: “No” (0 points), “Partially” (0.5 points) and “Yes” (1 point). The final score is calculated by summing the scores of all 25 items, multiplying it by 4 and then subtracting it from 100 to generate a percentage score between 0% to 100% (100% = no disability). Two missing responses are permitted [20]. Several studies demonstrated strong psychometric properties of the SFI, that includes good construct validity, internal consistency, reliability, responsiveness, and measurement error [20–26].

*The Oswestry Disability Index*. ODI was used to assess low back pain related disability. The ODI questionnaire includes measurement of 10 items: pain intensity, personal care, lifting, walking, sitting, standing, sleeping, sexual life, social life, and traveling. Each item is rated on a scale from 0 (no restriction due to LBP) to 5 (major restrictions due to LBP). ODI is scored by summing the items and multiplying them by 2. This number is considered as a percentage of the patient’s subjective disability [17].

*Numeric rating scale*. NRS is a pain measuring unidimensional tool. It is a subjective patient’s interpretation of the pain experience and assessment in a pain intensity scale from 0 to 10, where zero stands for ‘‘no pain” and maximum value stands for “highly intense pain” [16]. The NRS demonstrates positive and significant correlations with other measures of pain intensity and sensitivity to treatments that are expected to affect pain intensity [33].

Additionally, we collected key demographic and clinical information from the participants through a self-report method. Specifically, we gathered data on their age, as well as frequency and duration of low back pain experienced by the participants.

#### Sample size

Calculation of a minimum sample size for this validation study was based on the outcomes from the original study as well as other language validation studies. For an 80% likelihood of detecting differences allowing 15% attrition with p < 0.05, a minimum sample of 110 participants is required (reliability, n ≥ 45; concurrent criterion validity, n ≥ 106) [20,21,24–26]. For factor analysis, recommended minimum ratio is five participants per item [34]. The sample of this study and LBP characteristics are presented in Table 1.

**Table 1:** Study sample and characteristics of low back pain.

Characteristic	Cases (%)	Age (years) Mean [95%CI]	Pain duration (years) Mean [95%CI]	Pain frequency: less than 3 days/week (%)	Pain frequency: 3 or more days/week (%)
**Study sample**	125 (100%)	44.6 [42.1, 47.1]	7.0 [5.5, 8.5]	72 (59.5%)	49 (40.5%)
**Male**	38 (30.4%)	43.8 [39.6, 48.7]	10.2 [6.6, 13.9]	26 (21.5%)	10 (8.3%)
**Female**	87 (69.6%)	44.9 [41.7, 47.8]	5.6 [4.3, 7]	46 (38%)	39 (32.2%)

Abbreviations: %–percentage points, 95%CI– 95% Confidence Interval for Mean.

#### The pilot SFI-LT testing in a group of young subjects

The inclusion criteria for the SFI-LT testing were the same except the age between 18–45 years and working conditions–only office workers working in a sitting position at least 30 hours per week for not less than 1 year.

#### Statistical analysis

The data were analyzed using Statistical Package for the Social Sciences (SPSS) Version 27 Software for MacOS (IBM, Armonk, NY, USA).

Descriptive statistics were used to describe demographic data of the study population and pain duration. One-sample Kolmogorov-Smirnov test was used for normality testing. Data did not match normal distribution, therefore, a non-parametrical Wilcoxon and Mann-Whitney tests were applied for statistical analysis. Results are presented as means with their corresponding confidence interval, as well as in both absolute and percentage frequencies.

Corrected significance level set at p < 0.05 for all analyses.

#### Reliability analysis

For assessing reliability, our approach encompassed several dimensions. The internal consistency was determined by Cronbach’s α coefficient and item-total correlations. The Cronbach’s α values of 0.70–0.95 were considered of good internal consistency [35].

Test-retest reliability was performed using the intra-class correlation (ICC). All participants completed the SFI-LT twice with an interval of 3 to 7 days to assess reliability. Reliability was considered as good when ICC ≥ 0.70 [35]. We also incorporated the Spearman-Brown calculation to further assess the stability of our questionnaire over time; the value of 0.80 and above identified adequate construct stability, while 0.90 and above–good construct stability of the questionnaire [36].

In addition, measurement error was determined from the MDC 90 analysis [37]. Standard error of measurement (SEM) was calculated using the formula: $SEM=SD\sqrt{\left(1-r\right)},$ where SD is the standard deviation of the measurement and *r* is the reliability coefficient for the test and Pearson’s correlation coefficient between test and retest values.

#### Validity analysis

The validity of the questionnaire was assessed through several methods. Construct validity was determined by calculating the Spearman’s correlation ρ between the SFI-LT, ODI-LT and NRS. Correlation when 0.81–1.0 was considered as excellent, between 0.61–0.80 very good, between 0.41–0.60 good, between 0.21–0.40 fair, and between 0–0.20 poor [38]. All PRO measurements were performed during the same visit.

Additionally, the floor and ceiling effect which is described as a percentage of the sample achieving the highest or the lowest possible scores was evaluated. The percentage of maximum or minimum scores higher than 15% fails to meet standards [35].

Factor structure was analyzed using exploratory factor analysis (EFA). The loading suppression for the maximum likelihood extraction (MLE) was at 0.3 [39]. A-priori extraction requirements were as follows: scree plot inflection, Eigenvalue > 1.0 and variance > 10% [34].

Corrected significance level set at p < 0.05.

## Results

### Stage 1. Translation and cross-cultural adaptation

Translation process went smoothly without difficulties. Minor modifications were implemented to the formulation of some questions based on cultural relevance. We encountered the most difficulty translating the word “affected” (questions 9, 13, 16, 20) which in Lithuanian language may not be interpreted correctly if translated verbatim. A specialist in the Lithuanian language, T1, T2 translators and a bilingual professor in medicine agreed to accept T1 translation by choosing the phrase “more difficult.”

No patients reported problems or difficulties comprehending and completing the SFI-LT. There were no unanswered items in SFI-LT. As a result, the SFI was successfully translated into the Lithuanian language and cross-culturally adapted.

### Stage 2. Psychometric investigation

#### Results of PRO measurements

SFI-LT, ODI-LT and NRS results are presented in Table 2. The SFI-LT results are presented equated to ODI-LT (subtracted from 100). The percentage of disability was statistically significantly higher in SFI-LT questionnaire than in ODI-LT (z = –9.19, p < 0.001). There was no statistically significant difference between male and female in SFI-LT results (z = –1.28, p = 0.20), as well as in ODI-LT results (z = –1.10, p = 0.27). The Mean [95%CI] of SFI-LT scores in males was 28.3 [21.6, 35.9], in females 32.5 [28.2, 37.4], whereas the mean [95%CI] of ODI-LT scores in males was 12.7 [9.6, 16.1] and 14.8 [12.7, 17.3] in females.

**Table 2:** Results of all patient reported outcome measurements.

PRO tool (value)	Mean [95%CI]
**NRS (0–10)**	4.5 [4.2, 4.9]
**ODI-LT (0–100%)**	14.3 [12.5, 16.2]
**SFI-LT (0–100%)**	31.6 [27.8, 35.4]

Abbreviations: PRO–Patient Reported Outcome, NRS–numeric rating scale, ODI-LT–Oswestry Disability Index–Lithuanian version, SFI-LT–Spine Functional Index–Lithuanian version, 95%CI– 95% Confidence Interval for Mean.

#### Reliability analysis

*Internal consistency*. The SFI-LT internal consistency was excellent with Cronbach’s α = 0.92 (N = 125).

Item-total correlations ranged from 0.29 to 0.73 (Table 3).

**Table 3:** Reliability statistics for the Spine Functional Index.

Question	Item	Item-Total Correlations	α If Item Deleted	α
1	Stay at home	0.31	0.92	
2	Change positions	0.57	0.92	
3	Avoid heavy jobs	0.43	0.92	
4	Rest more often	0.57	0.92	
5	Ask others to do things for me	0.38	0.92	
6	Pain most of the time	0.5	0.92	
7	Difficulty while lifting and carrying	0.6	0.92	
8	Appetite is affected	0.31	0.92	
9	Affected walking, normal recreation, sport activities	0.69	0.91	
10	Difficulty with household and family duties	0.71	0.91	
11	Sleep less well	0.55	0.92	
12	Need of assistance with personal care	0.29	0.92	
13	Affected daily activity	0.6	0.92	
14	More irritable	0.48	0.92	
15	Feel weaker or stiffer	0.73	0.92	
16	Transportation independence is affected	0.56	0.91	
17	Get dressed more slowly	0.39	0.92	
18	Difficulty moving in bed	0.59	0.92	
19	Difficulty concentrating, reading	0.36	0.92	
20	My sitting is affected	0.48	0.92	
21	Difficulty getting in and out of chair	0.59	0.92	
22	Stand only for short periods of time	0.57	0.92	
23	Difficulty squatting, kneeling	0.66	0.91	
24	Difficulty reaching down	0.66	0.91	
25	Go upstairs slower or use a rail	0.72	0.91	
	Total			0.92

*Stability in time*. Test-retest correlation with 3 to 7 days interval showed good construct stability of the SFI-LT. The correlation (Spearman-Brown coefficient) between responses of the two surveys was 0.97.

*Test-retest reliability and measurement error*. The value of ICC for the entire group was 0.82 (CI ranged from 0.75 to 0.87) which represents good reliability. Measurement error from SEM was 6.96, from MDC was 16.24.

#### Validity analysis

*Construct validity*. Construct validity between the SFI-LT and ODI-LT was excellent (ρ = 0.83) and good between the SFI-LT and NRS (ρ = 0.55).

*Floor and ceiling effects*. The floor and ceiling effects were not detected in any of the PROM questionnaires. The minimum score was noted in 8 persons (6.4%) in the ODI-LT questionnaire and was not present in SFI-LT.

**Fig 2:**
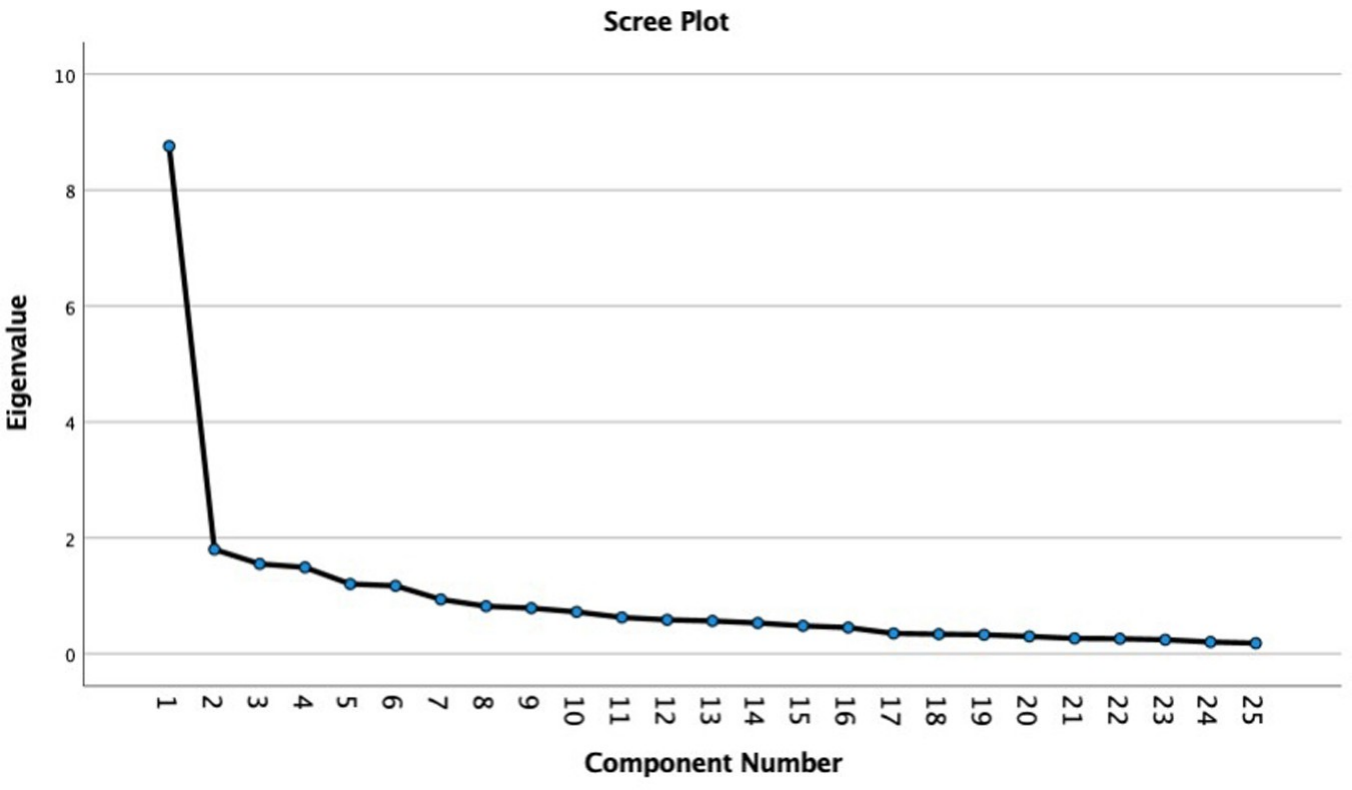
The scree plot supported a one-factor solution.

*Factor structure*. The correlation matrix for the SFI-LT was determined as suitable from the Kaiser-Meyer-Olkin (KMO = 0.87) and Bartlett’s Test of Sphericity (p < 0.001). In initial analysis, Eigenvalues for six factors were > 1, although only one factor accounted for more than 10% variance (35.04%) (Table 4). The Scree Plot inflexion occurred at the second point (Fig 2). The item loading for the one-factor solutions for the MLE method are shown in Table 5. Considering these three criteria, a unidimensional structure of the questionnaire is the most applicable.

**Table 4:** Cumulative variance explained by all the components.

Question	Initial Eigenvalues			Extraction Sums of Squared Loadings		
	Total	% of Variance	Cumulative %	Total	% of Variance	Cumulative %
1	8.759	35.037	35.037	8.759	35.037	35.037
2	1.803	7.210	42.248	1.803	7.210	42.248
3	1.552	6.207	48.454	1.552	6.207	48.454
4	1.492	5.969	54.423	1.492	5.969	54.423
5	1.205	4.821	59.245	1.205	4.821	59.245
6	1.174	4.697	63.942	1.174	4.697	63.942
7	.940	3.760	67.703			
8	.822	3.287	70.990			
9	.790	3.158	74.148			
10	.724	2.896	77.044			
11	.629	2.515	79.559			
12	.587	2.349	81.908			
13	.568	2.272	84.180			
14	.533	2.131	86.312			
15	.484	1.934	88.246			
16	.454	1.816	90.062			
17	.352	1.410	91.471			
18	.340	1.360	92.832			
19	.330	1.320	94.152			
20	.301	1.205	95.356			
21	.267	1.067	96.423			
22	.261	1.044	97.467			
23	.244	.975	98.442			
24	.205	.819	99.261			
25	.185	.739	100.000			

**Table 5:** Factor loading items for the one-factor solution.

Question	Item	Factor loading	Mean [95%CI]
1	Stay at home	.345	0.21 [0.2, 0.3]
2	Change positions	.464	0.62 [0.6, 0.7]
3	Avoid heavy jobs	.494	0.4 [0.3, 0.5]
4	Rest more often	.490	0.34 [0.3, 0.4]
5	Ask others to do things for me	.397	0.26 [0.2, 0.3]
6	Pain most of the time	.507	0.36 [0.3, 0.4]
7	Difficulty while lifting and carrying	.530	0.37 [0.3, 0.4]
8	Appetite is affected	.354	0.1 [0.1, 0.1]
9	Affected walking, normal recreation, sport activities	.595	0.4 [0.3, 0.5]
10	Difficulty with household and family duties	.615	0.34 [0.3, 0.4]
11	Sleep less well	.508	0.34 [0.3, 0.4]
12	Need of assistance with personal care	.462	0.04 [0.0, 0.1]
13	Affected daily activity	.580	0.12 [0.1, 0.2]
14	More irritable	.520	0.27 [0.2, 0.3]
15	Feel weaker or stiffer	.653	0.42 [0.4, 0.5]
16	Transportation independence is affected	.546	0.17 [0.1, 0.2]
17	Get dressed more slowly	.524	0.1 [0.1, 0.1]
18	Difficulty moving in bed	.613	0.22 [0.2, 0.3]
19	Difficulty concentrating, reading	.375	0.12 [0.1, 0.2]
20	My sitting is affected	.479	0.36 [0.3, 0.4]
21	Difficulty getting in and out of chair	.581	0.24 [0.2, 0.3]
22	Stand only for short periods of time	.514	0.3 [0.2, 0.4]
23	Difficulty squatting, kneeling	.622	0.36 [0.3, 0.4]
24	Difficulty reaching down	.576	0.36 [0.3, 0.4]
25	Go upstairs slower or use a rail	.672	0.31 [0.2, 0.4]

Abbreviations: 95%CI– 95% Confidence Interval for Mean.

### The pilot SFI-LT testing in young subjects

Eighty-seven subjects, 32 males (Mean [%95CI] of age 31.0 [28,7, 33.33] and 55 females (Mean [%95CI] of age 32.2 [30.43, 34.05] participated in the SFI-LT testing. The results of the SFI-LT, NRS and LBP duration are presented in Table 6. There were no statistically significant differences between gender and SFI-LT or NRS. Statistically significant difference was found in LBP duration between males and females.

**Table 6:** Results of the SFI-LT, NRS and LBP duration.

Characteristic	SFI-LT (0–100%) Mean [%95CI]	NRS (0–10) Mean [%95CI]	LBP duration (years) Mean [%95CI]
**All**	83.4 [80.1, 86.7]	2.3 [1.8, 2.7]	6.0 [4.6, 7.5]
**Male**	85.2 [79.6, 90.7]	2.1 [1.3, 2.8]	6.7 [4.1, 9.3]
**Female**	82.3 [78.1, 86.5]	2.4 [1.8, 3.0]	5.6 [3.9, 7.4]
**Z value**	-1.28	-2.19	-0.34
**P value**	0.2	0.03	0.73

Abbreviations: SFI-LT–Spine Functional Index–Lithuanian version, NRS–numeric rating scale, LBP–low back pain. 95%CI– 95% Confidence Interval for Mean, %—percentage points.

Out of all the subjects who evaluated LBP with zero points, 16 (69.6%) subjects scored 100% (no disability) on the SFI-LT, whereas 3 (13%) of them scored 98%, 2 (8.7%) scored 94% and 2 subjects (4.3%) scored 92% and 86% respectively.

Correlations of SFI-LT with NRS and duration of LBP were statistically significant (p < 0.05). Correlation between SFI-LT and NRS showed very good, (R = 0.68) relationship. Good relationship was found between SFI-LT percentage score and duration of LBP (R = 0.47).

## Discussion

The primary purpose of this study was to translate, cross-culturally adapt, and validate the SFI in the Lithuanian language. The SFI-LT proved to be valid and reliable questionnaire with a good internal consistency for evaluation of functional status in low back pain patients. The original version of the SFI was created in English [20]. This questionnaire was cross-culturally adapted and validated in several languages and cultures: Spanish, Turkish, Korean, Chinese, Persian and Polish [21–26]. Cronbach’s alpha is designed to assess the internal consistency of a scale, which is expressed as a number between 0 and 1. Internal consistency refers to the interrelationship of all the test items, as all the test items measure the same concept. The acceptable values of alpha are ranging from 0.70 to 0.95 [40]. Comparing our results with other studies, the Cronbach’s α in our study represented perfect internal consistency and was slightly higher α = 0.92 (0.91–0.92) than in other studies, e.g., English α = 0.91 [20], Spanish α = 0.85 (0.80–0.88) [21], Turkish α = 0.85 (0.80–0.85) [26], Persian α = 0.80 (0.78–0.82) [25], Chinese α = 0.91 (0.80–0.95) [22], Korean α = 0.88 [23], and Polish α = 0.90 (0.70–0.95) [24].

Corrected item-total correlations in this study ranged from 0.29 to 0.73, that indicates good discrimination of the questions. Similar results were found in previous studies: corrected item-total correlations in the Chinese study ranged from 0.31 to 0.69 and from 0.28 to 0.58 in the Persian study.

Although the test-retest reliability value of ICC over 0.70 is considered excellent [35], results in our study (ICC = 0.82) were lower than in other studies: English ICC = 0.98 [20], Spanish, Chinese and Persian, where ICC = 0.96 [21,22,25], also Turkish ICC = 0.93 [26], Korean ICC = 0.94 [23] and Polish ICC = 0.97 [24]. It is worth noting that participants in our study had chronic non-specific LBP, which usually is more stable for one week periods than acute pain, therefore higher ICC result could be predicted. Lower ICC result in our study could be explained by the influence of a more homogenous group of subjects as already noticed by other authors [41]. It might be influenced by the cultural specifics, re-test period timing (in English study the second examination was performed on day 3 only), or differences in sample sizes (in our study all 125 participants completed the SFI-LT for a second time, whereas in Persian version a subgroup of 31 participants, in Spanish study– 51 participants completed SFI for the second time).

In our study, Spearman-Brown coefficient of the SFI-LT was 0.97, that means good construct stability, nevertheless the SFI-LT demonstrated higher error values (SEM = 6.96 and MDC = 16.24%) than the previous studies [20,21,24–26]. This could be associated with a high variation in the SD of baseline presenting scores, cultural or geographic specifics of the sample.

Results of our study demonstrated excellent construct validity between the SFI-LT and ODI-LT (ρ = 0.83) and good between the SFI-LT and NRS (ρ = 0.55). SFI and ODI both evaluate pain related functional restriction, while NRS is a pain evaluation scale, therefore higher correlation between SFI and ODI could be expected. We found three studies using ODI questionnaire for evaluation of construct validity. In the Chinese study correlation was good (ρ = 0.58), in Turkish–very good (ρ = 0.71), in Polish–excellent (ρ = |0.82|). In Persian, Turkish, and Korean studies the correlations of SFI with another PRO assessment tool–Roland-Morris questionnaire were presented as follows: ρ = 0.64 (very good), ρ = 0.58 (good) and 0.75 (very good). In the original study as well as in the Turkish and Korean studies a different back pain associated functional scale–the Functional Rating Index (FRI) was used. In the original study the correlation of the SFI with FRI was excellent (ρ = 0.85), in other studies the correlation was good, i.e., ρ = 0.60 in the Turkish study and ρ = 0.57 in the Korean study.

Participants in our study were experiencing chronic low back pain, which generally affects functional status in everyday activities. If the floor effect would be exhibited, that might mean that the questionnaire is not sensitive enough in detecting the limitations of functional status and the data cannot score below the limit. On the contrary, ceiling effect means that the measurement cannot exceed the limit. Results of our study as well as previous studies [20–26] presented no floor or ceiling effects of the SFI. A total value of 0% of ODI-LT questionnaire score in our study occurred in 6.4% of cases thus cannot be considered as a floor effect as it presented in less than 15%. The appearance of floor effects of ODI was noted in a few previous research studies [42–44].

Exploratory factor analysis was chosen based on experience of previous studies using it when the sample is insufficient for confirmatory factor analysis. Using exploratory factor analysis, six factors were identified that explains 63.94% of variance. The results of this study supported previous ones; one-factor structure was the most reliable with the SFI questionnaire, as only one factor had a variance of more than 10% [21,25,26]. All items have sufficient item loadings presenting a solid one-factor structure.

We also tested the final version of SFI-LT in young people with mild to moderate or no pain. We investigated young office workers who are sitting for long periods and frequently complain about low back problems. The results showed that SFI-LT is a purposeful tool for evaluation of functional status affected by LBP in young people with not severe pain. Results of this study demonstrated very good relationship between NRS and SFI-LT. Nevertheless, in 31.4% of all young subjects SFI-LT could detect functional limitations in daily life activities due to the spine problems although they scored LBP as 0 on NRS.

### Study limitations and strengths

Our study was limited to evaluation of low-back patients only and lacked patients with pain in other regions of the spine. To our knowledge, there are no valid, translated, and cross-culturally adapted questionnaires into Lithuanian language for cervical functional status evaluation (e.g., Neck Disability Index), or other similar functional status-related tools, such as Roland-Morris questionnaire, Quebec Back Pain Disability Scale or Functional Rating Index. The ODI is mostly targeted for evaluation of functional disability secondary to LBP [17], therefore we were constrained to the possibility of studying patients with LBP.

In our study’s methodology for assessing test-retest reliability, we followed a protocol consistent with other validation studies of the SFI, where retesting was performed using only the SFI questionnaire itself [21,25,26]. We did not inquire directly about changes in patients’ conditions between the initial and subsequent administration of the questionnaire. This approach relies on the assumption that within the short timeframe between tests (3 to 7 days), significant clinical changes in a chronic condition such as non-specific low back pain are unlikely. However, we acknowledge that this does not account for the possibility of acute fluctuations in symptoms or changes in patient condition that could occur within the retest interval.

Furthermore, confirmatory factor analysis (CFA) usually is used for factor structure clarification. For CFA, sample size must be five to ten times bigger than with EFA; this would require at least 400 participants for 25-item questionnaire clarification of factor structure [41,45]. This was beyond the scope of this study and thus it was not performed. The strengths of this study include the use of standardized methods for cross-cultural adaptation and psychometric investigation.

### Future considerations

Future research should focus on a broader evaluation of the SFI-LT, particularly in relation to specific spinal regions and in diverse patient groups, such as those recovering from spine surgery or experiencing acute pain. It’s also crucial to assess how well the SFI-LT can monitor patient changes over time, which is essential for understanding its effectiveness in showing treatment impacts for back pain.

Enhancing the test-retest reliability of the SFI-LT in future studies is another key area. This can be achieved by directly asking about the stability of the patient’s condition. Additionally, implementing supplementary methods, like a brief interview or a follow-up questionnaire, could effectively track any interim changes in symptoms or functional status. These methods would specifically aim to identify any significant clinical changes since the initial assessment.

Furthermore, it is important to explore the application of the SFI-LT across different sociodemographic groups.

## Conclusions

The newly translated and validated SFI-LT is a new reliable and valid PRO instrument for functional evaluation of back pain in Lithuanian speaking patients. It incorporates supportive psychometric values of validity, reliability, internal consistency, and factor structure. The results of this study are comparable with the results of the original study and with other SFI validation and cross-cultural adaptation studies.

## Supporting information

S1 File SFI-ODI data. (ZIP)

S2 File Lithuanian version of the Spine Functional Index. Modified from [16] under a CC BY license, printed with permission from Elsevier, original copyright 2019.
(PDF)
