# Supplementary material for: Cross-cultural adaptation and validation of the Lithuanian version of the Spine Functional Index
Source: PLoS One. 2024 Mar 13;19(3):e0299719. doi: 10.1371/journal.pone.0299719 (PMC10936852; doi:10.1371/journal.pone.0299719)
Supplement: S2 File — Modified from [16] under a CC BY license, printed with permission from Elsevier, original copyright 2019. (PDF) [file pone.0299719.s002.pdf]

STUBURO FUNKCIJOS INDEKSAS (SFI)

DATA: ____________________

VARDAS, PAVARDĂ: ____________________ **PAŽAIDA:** ____________________ ☐ **Kaklas** ☐ **Vidurină nugaros dalis** ☐ **Apatină nugaros dalis**

PRAĠOME UĢPILDYTI. Dăl jî sÔ stuburo bî klăs jums gali bî ti sunku atlikti tam tikrus Ţprastus veiksmus. Ġiame sŃraġe pateikti teiginiai, kuriais paprastai save apibūdina su tokiais sunkumais susidūrę žmonės. Pagalvokite apie savo kelias pastarŃsias dienas. **Jei su teiginiu sutinkate, pažymėkite langelį „Iš dalies“ arba „Taip“. Jei su teiginiu nesutinkate, pažymėkite langelį „Ne“.**

DĂL STUBURO BŪKLĖS:

Ne	Iġ dalies	Taip	
☐	☐	☐	1. Aš didġiŃjŃ dalĮ laiko bî nu namuose.
☐	☐	☐	2. Aš daġnai keiļ iu padătĮ, kad bî tÔ patogu.
☐	☐	☐	3. Aš vengiu sunkiÔ fiziniÔ darbÔ (pvz., valymo, didesniÔ nei 5 kg svoriÔ kėlimo, sodo darbÔ ir kt.).
☐	☐	☐	4. Aš daġniau ilsiuosi.
☐	☐	☐	5. Aš praġau kitÔ, kad uġ mane atliktÔ tam tikrus darbus.
☐	☐	☐	6. Aš beveik visŃ laikŃ jauļ iu skausmŃ / problemŃ.
☐	☐	☐	7. Man sunku kelti ir neġti svorius (pvz., krepġius, pirkinius, sverianļ ius iki 5 kg).
☐	☐	☐	8. Pasikeită mano apetitas.
☐	☐	☐	9. Man yra sunkiau vaikġļ ioti, uġsiimti laisvalaikio veikla ar sportuoti.
☐	☐	☐	10. Aš susiduriu su sunkumais atliekant įprastus namų ruošos darbus ar su šeima susijusias pareigas.
☐	☐	☐	11. Aš blogiau miegu.
☐	☐	☐	12. Man reikia pagalbos pasirūpinant savo asmens priežiūra (pvz., maudantis ir rūpinantis higiena).
☐	☐	☐	13. Man yra sunkiau atlikti kasdienes veiklas (darbą, palaikyti socialinį ryšį).
☐	☐	☐	14. Aš esu irzlesnis (-ė) ir (ar) prastesnės nuotaikos.
☐	☐	☐	15. Aš jaučiuosi silpnesnis (-ė) ir (ar) labiau sustingęs (-usi).
☐	☐	☐	16. Man yra sunkiau naudotis transportu (vairuoti, naudotis viešuoju transportu).
☐	☐	☐	17. Man reikia pagalbos rengiantis arba aš rengiuosi lėčiau.
☐	☐	☐	18. Man sunku judėti lovoje.
☐	☐	☐	19. Man sunku susikaupti ir (ar) skaityti.
☐	☐	☐	20. Man yra sunkiau sėdėti.
☐	☐	☐	21. Man sunku atsisėsti ant kėdės ir nuo jos atsistoti.
☐	☐	☐	22. Aš galiu stovėti tik trumpą laiko tarpą.
☐	☐	☐	23. Man sunku pritūpti ir (ar) atsiklaupti.
☐	☐	☐	24. Man sunku pasilenkti (pvz., pakelti daiktus, užsimauti kojines).
☐	☐	☐	25. Aš lėčiau lipu laiptais arba lipant naudojuosi turėklais.

SFI REZULTATAS. Aukščiau pateiktos dalies rezultatui nustatyti sudėkite pažymėtus langelius:

☐ **IŠ VISO (SFI balai)** **100 skalė: 100 – (IŠ VISOx4) =** ☐ **%**

Min. aptinkamas pokytis (90% PI): Kaklas = 6,9 % ar 1,7 SFI balai; **Vidurinė ir apatinė nugaros dalis** = 5,9 % ar 1,5 SFI balai; **Visas stuburas** = 6,5 % ar 1,6 SFI balai Mažesnis pokytis, nei nurodytas, gali atsirasti dėl paklaidos.
